# Accelerating Evidence Synthesis: A BERT-Assisted Workflow for Meta-Analyses of Radiotherapy Complications in Nasopharyngeal Carcinoma

**DOI:** 10.3390/reports9010090

**Published:** 2026-03-18

**Authors:** Tsair-Fwu Lee, Wen-Ping Yun, Hung-Wei Hsu, Jyun-Jie Wu, Ya-Shin Kuan, Yi-Lun Liao, Cheng-Shie Wuu, Liyun Chang, Yang-Wei Hsieh, Pei-Ju Chao

**Affiliations:** 1 Medical Physics and Informatics Laboratory of Electronic Engineering, National Kaohsiung University of Science and Technology, No. 415, Jiangong Rd., Sanmin Dist., Kaohsiung 80778, Taiwan; tflee@nkust.edu.tw (T.-F.L.); 1106405106@nkust.edu.tw (W.-P.Y.); f111152133@nkust.edu.tw (H.-W.H.); f112152118@nkust.edu.tw (J.-J.W.); kwanbeardad724@gmail.com (Y.-S.K.);; 2 Graduate Institute of Clinical Medicine, Kaohsiung Medical University, Kaohsiung 80708, Taiwan; 3 Department of Medical Imaging and Radiological Sciences, Kaohsiung Medical University, Kaohsiung 80708, Taiwan; 4 Cross Grace Dental Clinic, Kaohsiung 80708, Taiwan; 5 Department of Radiation Oncology, Columbia University, New York, NY 10032, USA; csw6@columbia.edu; 6 Department of Medical Imaging and Radiological Sciences, I-Shou University, Kaohsiung 82445, Taiwan; cliyun2000@gmail.com; 7 Department of Radiation Oncology, Kaohsiung Veterans General Hospital, Kaohsiung 80708, Taiwan; 8 Department of Radiation Oncology, Kaohsiung Chang Gung Memorial Hospital, Chang Gung University College of Medicine, Kaohsiung 80708, Taiwan

**Keywords:** meta-analysis, nasopharyngeal carcinoma, complications, BERT, natural language processing, artificial intelligence, radiotherapy

## Abstract

**Background/Objectives**: This study developed and evaluated a BERT-assisted literature screening workflow to support meta-analyses of postradiotherapy complications in nasopharyngeal carcinoma patients. The aim was to automate key screening steps to improve downstream screening efficiency and consistency, while minimizing time and bias during manual reviews. **Materials and Methods**: A bidirectional encoder representations from transformers (BERT) model was integrated into a standard systematic review pipeline for studies on postradiotherapy complications in nasopharyngeal carcinoma. The workflow combined automated BERT-based classification with manual verification and followed PRISMA and PICOS guidelines for literature identification, screening, and eligibility assessment. Model training involved hyperparameter tuning and comparison of different optimizers to maximize screening performance against a manually curated reference set, with particular attention to discrimination (AUC) and processing time. **Results**: From an initial corpus of 6496 records, the combined automated and manual workflow identified 23 eligible studies for meta-analysis. The included studies showed substantial heterogeneity (I^2^ = 86.85%), supporting the use of a random-effects model to pool outcomes. The BERT model optimized with an Adagrad optimizer achieved an AUC of 0.77 for relevant-study classification and reduced screening time to 1142 s. To demonstrate the workflow’s utility, a downstream meta-analysis was conducted using the identified studies. As a downstream application based on the identified studies, a quantitative synthesis was conducted, in which (meta-analysis of the 23 included studies), a random forest model—evaluated across those studies—achieved an AUC of 0.92 under a fixed-effect analysis for predicting postradiotherapy complications. **Conclusions**: Integrating BERT into the literature screening phase of meta-analysis for postradiotherapy nasopharyngeal carcinoma complications markedly improved screening efficiency while maintaining acceptable classification performance. This workflow demonstrates the feasibility of transformer-based assistance for systematic reviews and provides a foundation for developing disease-specific, AI-augmented evidence synthesis pipelines in oncology.

## 1. Introduction

In recent years, the body of literature on nasopharyngeal carcinoma (NPC) has experienced significant growth, reflecting heightened global interest and advancing research in this field. In the big data era, efficiently synthesizing vast literature under time constraints is increasingly challenging, particularly for NPC where diverse factors and inconsistent methodologies across studies hinder clear delineation of relationships among findings [1].

To address these complexities, systematic reviews have traditionally aggregated and synthesized multiple studies into consolidated viewpoints, but they are limited by subjective interpretations and non-universal methodological choices. This has led to meta-analyses, which enhance systematic reviews with robust statistical analysis for greater validity, reliability, and objectivity [2,3,4]. Yet, meta-analyses remain labor-intensive and time-consuming—often requiring up to 463 days [5]—creating bottlenecks in large-scale datasets, especially in oncology where data complexity and rapid literature growth demand scalable, objective approaches.

Advancements in artificial intelligence, particularly natural language processing (NLP), offer transformative solutions. For instance, Feng et al. [6] showed that machine learning models, including NLP-based ones, significantly boost literature screening efficiency in medicine.

Among NLP advancements, GPT-based models like GPT-3 and GPT-4 excel in generative tasks, coherent text production, query answering, and data synthesis, aiding automated summarization and medical insights. However, their scalability involves high computational demands, and they may underperform in classification relative to BERT due to unidirectional architecture and resource intensity [7].

In contrast, the BERT (bidirectional encoder representations from transformers) model stands out for its pretrained architecture that captures bidirectional context, enabling accurate identification and extraction of relevant studies from vast amounts of literature [8,9,10]. BERT’s strength in understanding and extracting information—unlike GPT’s focus on generation—aligns ideally with literature screening for meta-analyses.

This study has two main objectives: (1) to develop and optimize a BERT-assisted workflow for automated literature screening; (2) to demonstrate the workflow’s practical application by conducting a meta-analysis of post-radiotherapy complications in NPC.

Unlike prior BERT-based screening approaches that primarily emphasize record-level classification performance, this study focuses on a workflow-level framework that links model optimization, deployment-stage efficiency, and downstream evidence synthesis.

## 2. Materials and Methods

### 2.1. Data Annotation and Screening Workflow

A total of 6496 titles and abstracts were retrieved from PubMed and Web of Science. A domain expert annotated an initial subset of records according to predefined PICOS-based eligibility criteria to establish the gold standard for model training. The dataset was subsequently divided into training (80%), validation (10%), and test (10%) sets. To account for class imbalance, stratified sampling was applied during dataset splitting to ensure that relevant studies were proportionally represented across the training, validation, and test sets.

The trained BERT model was applied as a digital screener to perform large-scale pre-screening, filtering out obviously irrelevant studies (e.g., animal studies, non-radiotherapy studies, and non-nasopharyngeal carcinoma publications). Records flagged as potentially relevant were subjected to full-text review by the same domain expert, who made the final eligibility determination for inclusion in the meta-analysis.

The BERT model was used exclusively for literature screening and did not train, modify, or influence any clinical prediction models. The subsequent meta-analysis summarizes predictive-model performance as reported in the finally included studies and serves solely as a downstream application enabled by study identification.

### 2.2. Operational Definitions of Study Relevance

Studies were defined as relevant if they met the predefined PICOS-based criteria, specifically if they were primary research articles reporting predictive models for post-radiotherapy complications in patients with nasopharyngeal carcinoma and provided quantitative model performance metrics. Studies were considered irrelevant if they did not meet one or more of these criteria, including review articles, animal studies, editorials, studies unrelated to nasopharyngeal carcinoma or radiotherapy, or studies lacking quantitative performance measures.

### 2.3. Research Framework

**Figure 1:**
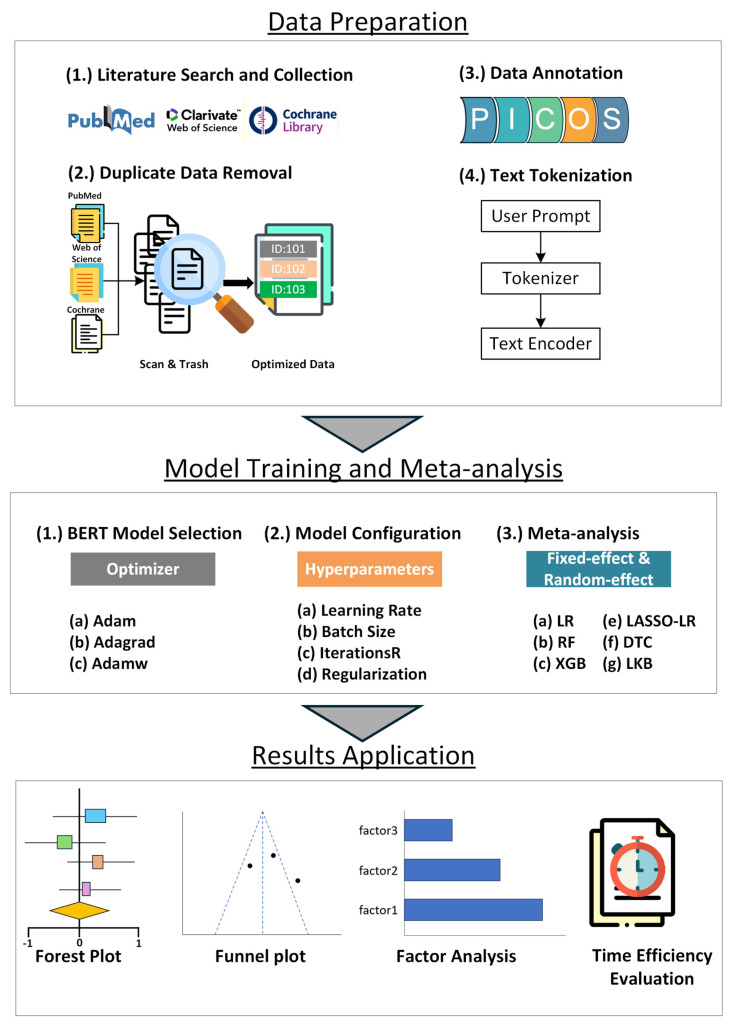
**Research workflow diagram.** Abbreviations: BERT, Bidirectional Encoder Representations from Transformers.

This study utilizes the BERT model to optimize the literature screening process for postradiation therapy complications in nasopharyngeal carcinoma patients. The research process is divided into three main stages, data preparation, model training and meta-analysis, and results application, as depicted in Figure 1.

**Data Preparation Stage:** Relevant literature pertaining to nasopharyngeal carcinoma after radiation therapy was initially collected from scientific databases, such as PubMed and Web of Science. A custom Python script automates the removal of duplicate data. Detailed annotations and text tokenization are then performed to prepare a clean and structured dataset for subsequent analysis. The detailed Python data processing code and corresponding pseudocode are included in Supplementary Figure S1 for enhanced reproducibility.

**Model training and meta-analysis stage:** The BERT architecture was selected for its robust capacity to capture bidirectional contextual nuances. The research team meticulously adjusts the learning rates and other critical model parameters to optimally suit the research needs. The learning rate was initially set at 0.001 and adjusted during training to optimize performance on the dataset. The batch size was set at 5 with 300 training iterations to ensure model convergence. Additionally, L1 regularization was employed to reduce overfitting by penalizing the absolute values of weights, encouraging sparsity in the model’s parameters and enhancing its ability to generalize to unseen data.

**Results Application Stage:** Following literature screening, the identified studies were synthesized using standard meta-analytic techniques. Forest plots and funnel plots were used to summarize reported outcomes across studies. Separately, the time efficiency of the BERT-assisted screening workflow was evaluated by comparing screening time with conventional manual screening procedures.

### 2.4. BERT-Assisted Literature Screening Workflow

#### 2.4.1. Literature Search and Collection

**Figure 2:**
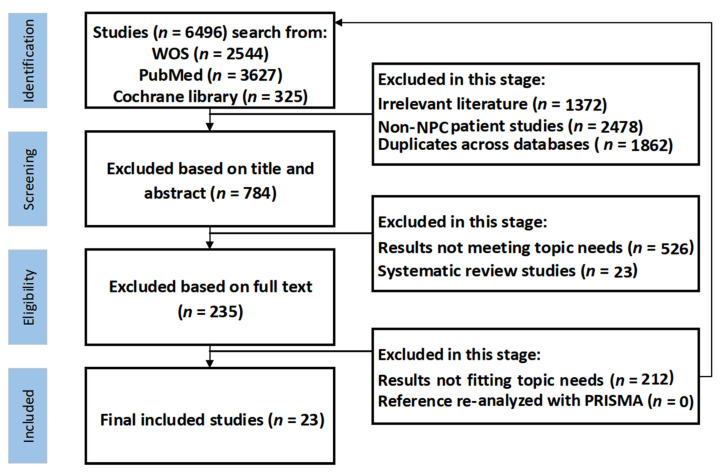
**Literature search flowchart.** Abbreviations: NPC, nasopharyngeal carcinoma; WOS, Web of Science; PRISMA, Preferred Reporting Items for Systematic reviews and Meta-Analysis.

As illustrated in Figure 2, the literature search and screening process uses the PRISMA method to ensure the high relevance and accuracy of the documents [11,12]. Beginning with 6496 documents, the process meticulously narrows down to 23 studies that meet stringent research standards.

**Search and collection phase:** Initial searches were conducted in databases, including Web of Science, PubMed, and the Cochrane Library. This phase often involves filtering through a vast volume of literature, necessitating an effective system to ensure the relevancy and accuracy of the selected documents.

**The screening stages include the following:**

**The initial** screening focused on removing duplicates and significantly irrelevant documents directly from the search results.

**Title and Abstract Screening:** Further refine the selection on the basis of titles and abstracts, targeting documents specifically related to postradiotherapy complications and excluding systematic reviews.

**Full Text Review:** Ensures that documents containing relevant AUC results are retained. Additional searches within the references were conducted to capture any potentially overlooked significant literature.

#### 2.4.2. Duplicate Data Removal

When multiple literature databases are selected, duplicate documents inevitably appear. A Python script is employed to automate the removal of duplicates, enhancing the process efficiency and ensuring a clean dataset for analysis.

#### 2.4.3. Model Selection and Configuration

BERT, renowned for its bidirectional training feature, is selected for its robust performance in text analysis [13,14]. The learning rate and other parameters are determined experimentally, optimizing model performance with strategies such as Adam, AdamW, and AdaGrad optimizers, which are considered for their adaptive learning rate adjustments [15,16,17].

### 2.5. Downstream Quantitative Synthesis (Meta-Analysis)

The outcome synthesized in this meta-analysis was defined as the study-level AUC reported for each predictive model, rather than patient-level clinical outcomes.

Heterogeneity among the included studies was assessed to account for differences in study populations, data sources, and modeling strategies. The I^2^ statistic and Cochran’s Q test were used to quantify between-study variability, and a random-effects model was adopted accordingly

#### Meta-Analysis Statistical Methods

All statistical analyses were conducted using Python (version 3.10.18). Meta-analytic calculations and heterogeneity analyses were implemented using the NumPy, SciPy, and statsmodels libraries. Forest plots and funnel plots were generated using matplotlib and pandas.

Quantifying heterogeneity via I^2^ statistics and Q tests helps validate the variability and credibility of the study findings, which are crucial for deriving accurate meta-analytical conclusions [18,19,20].

## 3. Results

**Time efficiency evaluation:** Table 1 presents comparative results of BERT models using different optimizers, focusing on their impact on performance and time efficiency. The Adagrad optimizer enabled the BERT model to achieve the highest AUC value of 0.770, attributed to its high adaptability to diverse datasets, allowing for optimal parameter adjustments on the basis of the variability of the data. Conversely, the Adam optimizer achieved an AUC of 0.664, while AdamW recorded the lowest value at 0.590, with its performance affected by its lower adaptability to the dataset than Adagrad.

In terms of time efficiency, Adagrad not only outperformed in AUC but also excelled in execution time, completing tasks in 1142 s—marginally quicker than AdamW’s 1159 s and Adam’s 1210 s. The BERT-assisted workflow substantially reduced the marginal screening time required during the deployment phase when compared with manual review, while initial setup costs for data annotation and model training remained necessary.

To avoid ambiguity regarding task scope, we note that the BERT model was used exclusively for literature screening (i.e., classifying records as relevant or irrelevant for eligibility assessment). It does not train, modify, or influence any clinical prediction models. The following quantitative synthesis summarizes predictive-model performance as reported in the finally included studies and represents a downstream application enabled by study identification, rather than an outcome of the screening classifier itself.

### Heterogeneity Analysis (Forest Plot)

**Figure 3:**
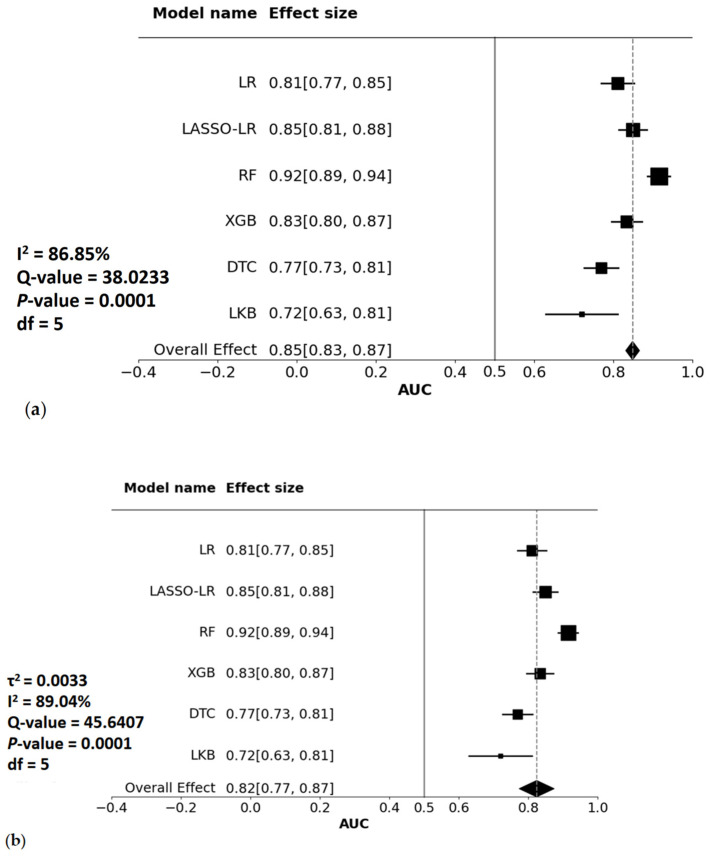
**Forest plots summarizing study-level AUC values reported for different types of clinical prediction models across the included studies. Each square represents the AUC reported for a specific predictive model within an individual study, with horizontal lines indicating the corresponding 95% confidence intervals. Diamonds indicate the pooled AUC estimates derived from meta-analysis. Panel (a) shows results under a fixed-effect model, and panel (b) shows results under a random-effects model.** Abbreviations: AUC, area under the ROC curve; Tau^2^, between-study variance; I^2^, heterogeneity index; Q-value, Cochran’s Q statistic value; *p* value, probability value; df, degrees of freedom; LR, logistic regression; LASSO, least absolute shrinkage and selection operator; RF, random forest; XGB, extreme gradient boosting; DTC, decision tree classifier; LKB, Lyman–Kutcher–Burman. **Notes:** The forest plot illustrates the AUC values and 95% confidence intervals for various predictive models. Each square represents the mean AUC for a specific model, with the size of the square reflecting the weight of the model in the analysis. The horizontal lines indicate confidence intervals. The diamond at the bottom represents the pooled AUC estimate derived from meta-analysis, with its width reflecting the corresponding confidence interval. Panel (**a**) shows the fixed-effect summary, whereas panel (**b**) shows the random-effects summary accounting for between-study heterogeneity. However, owing to the high heterogeneity observed (**I^2^ = 86.85%**, Q = 38.0233, *p* < 0.0001), a **random-effects model** (**b**) was used. This model accounts for variability between studies (τ^2^ = 0.0033, I^2^ = 89.04%), providing a more reliable overall effect estimate (**AUC = 0.82**) than the fixed-effects model (**AUC = 0.85**). High heterogeneity suggests significant differences between models due to variations in study design, populations, or methodologies. The random-effects model ensures robust and generalizable conclusions by accounting for this variability.

Heterogeneity Analysis (Forest Plot): Forest plots are used to display the results of heterogeneity analysis, where each entry represents the study-level AUC and corresponding confidence interval as reported in the included studies. Figure 3a,b shows that the best model result, with an AUC of 0.92, was for the random forest model with high heterogeneity (*p* value less than 0.05). In Figure 3b, the low τ^2^ value indicates that the differences between groups are small, which is why the results of the fixed-effect model and the random-effects model are similar. The I^2^ statistic also indicates high heterogeneity, as the value falls within the 75–-100% range. The high I^2^ value of 86.85% indicates substantial variability across studies, likely due to differences in designs, populations, and methodologies. To address this, the random-effects model accounts for between-study variability (τ^2^), providing a more appropriate pooled AUC estimate that reflects heterogeneity across studies.

**Figure 4:**
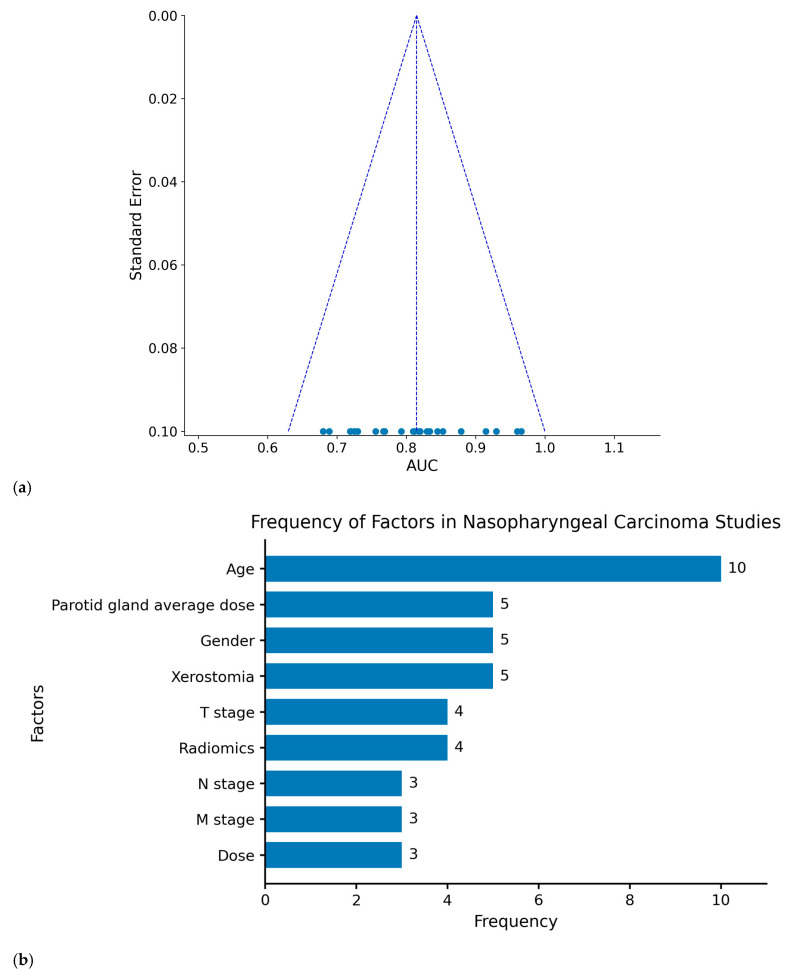
Comprehensive analysis of the included studies: (**a**) funnel plot of the area under the curve (AUC) and (**b**) frequency of predictors reported in the studies. In the funnel plot, the x-axis represents the AUC values and the y-axis represents the standard error of the AUC. **Blue dots represent individual studies**, and the dashed lines indicate the 95% confidence interval limits forming the expected funnel-shaped region. A symmetrical distribution of the studies suggests the absence of substantial publication bias.

**Publication bias analysis (funnel plot):** Figure 4 displays a funnel plot used to assess publication bias among studies in a meta-analysis. The vertical axis represents the standard error, a measure of the uncertainty of the study results, whereas the horizontal axis represents the AUC value, which is used to demonstrate the performance of the model. In the funnel plot, each point represents the result of a study, with higher AUC values indicating better predictive accuracy of the model. Ideally, if there is no publication bias, these points should be symmetrically distributed around the highest point. The majority of the points in the chart are concentrated around an AUC of approximately 0.8 and are quite tightly clustered, indicating very little variation and extremely low standard error, with no apparent publication bias. The results of the included studies are thus relatively stable.

**Factor analysis:** Figure 4b illustrates the frequency of various factors identified in studies related to nasopharyngeal carcinoma. “Age” was the most frequently occurring factor, appearing in 10 studies, underscoring its importance in assessing complications following radiotherapy in patients. “Parotid gland average dose” and “gender” each appeared in 5 studies, highlighting their importance as well. Other critical factors include “T stage,” “Xerostomia,” and “Radiomics,” which are essential for understanding and predicting different postradiotherapy complications. Notably, the inclusion of “Radiomics” in recent research underscores its emerging relevance and the trend toward its increased incorporation into future studies.

## 4. Discussion

To our knowledge, this is the first study to present an end-to-end BERT-assisted workflow specifically tailored to evidence synthesis on post-radiotherapy complications in NPC, integrating AI-assisted screening with meta-analytic synthesis within a single reproducible pipeline. The central contribution of this submission is the integration of a supervised transformer-based screening model with a conventional meta-analytic framework to manage a rapidly expanding literature while preserving interpretability for downstream clinical translation. In our analysis, the random forest model showed strong discriminatory performance (AUC = 0.92) for complication risk assessment, and our synthesis highlighted frequently reported clinical and dosimetric predictors (e.g., mean parotid dose and T-stage) that are consistent with established radiobiological and clinical considerations in NPC radiotherapy. Importantly, these performance metrics were extracted from the included studies and summarized as part of a downstream quantitative synthesis, rather than being outcomes generated by the BERT-based screening model itself.

A substantial level of heterogeneity was observed (I^2^ = 86.85%), which is more plausibly attributable to genuine clinical and methodological diversity across NPC studies—such as dose prescriptions, concurrent systemic therapy, biomarker definitions, follow-up duration, and complication ascertainment—than to sampling error alone. Therefore, we used a random-effects model to estimate a pooled effect that explicitly accounts for between-study variability, rather than assuming a single common effect size. Importantly, high heterogeneity implies that pooled estimates should be interpreted as context-dependent summaries. Accordingly, the pooled AUC should be viewed as a descriptive summary of reported model performance across heterogeneous studies, rather than as an estimate of a single underlying clinical effect. This reinforces the need for transparent selection criteria and reproducible screening procedures to avoid compounding bias through inconsistent inclusion decisions.

A key practical advantage of our workflow is the marked reduction in screening time (99.6% compared with manual screening), addressing a major bottleneck in systematic reviews and meta-analyses. The reduction was estimated by comparing total manual screening time (per-record review time × number of records) with the runtime of the automated pipeline. This estimate reflects marginal efficiency gains during the deployment stage of the workflow and does not account for the initial manual effort required for data annotation and model preparation. This finding aligns with recent evidence that AI-assisted screening can compress review timelines substantially. For example, recent evaluations of LLM-assisted screening have shown that review timelines may be condensed from months to hours, while also highlighting that sensitivity can vary depending on workflow design choices such as task framing and prompting [21]. Consistently, a human–LLM collaborative strategy that incorporates human verification has been shown to reduce annotation workload by approximately 80% while maintaining high reliability, supporting the broader principle that human oversight remains essential for safety-critical screening tasks [22]. In this context, our results suggest that a supervised BERT-based architecture remains a competitive and efficient option for binary relevance classification when labeled training data are available and the task definition is stable. Compared with generative LLM approaches, a supervised classifier may offer stronger controllability and reproducibility across iterations.

Our screening model achieved an AUC of 0.77 for relevance classification, and this should be interpreted in light of the intrinsic difficulty of literature screening. Screening tasks are typically characterized by severe class imbalance, where truly relevant records represent only a small subset of retrieved citations. Under such conditions, relying on single summary metrics (e.g., overall accuracy, or even AUC alone) can be insufficient to characterize practical screening safety. In particular, meta-analytic evidence across ML-based language model applications has emphasized that overall accuracy can be misleading in imbalanced classification settings and has recommended standardized reporting including confusion matrices and class-wise precision/recall/F1 scores to support reliable comparisons across studies [23]. Accordingly, we recommend that screening performance be reported with recall-focused metrics alongside AUC, and that the operational workflow prioritize sensitivity with targeted human verification.

Although our application is not radiology report mining, the radiology NLP literature provides a useful reference domain for what is achievable with transformer-based methods in clinically complex text; however, these tasks differ from literature screening and should not be interpreted as direct performance benchmarks. A systematic review of BERT applications in radiology has highlighted that transformer-based approaches are frequently used for classification and information extraction from free-text clinical reports, underscoring the value of bidirectional context modeling in medical language understanding [24]. Moreover, pooled evidence from a systematic review and meta-analysis of NLP models for information extraction from free-text radiology reports has reported high overall performance (e.g., sensitivity ~91%, specificity ~96%, AUROC ~0.98) while also noting the importance of heterogeneity and external validation in clinical NLP deployment [25]. These findings support two implications relevant to our study: transformer-based approaches can perform strongly when tasks are well-defined and data are representative, yet heterogeneity and generalizability concerns frequently constrain transferability. This further justifies the need for external validation and transparent reporting when translating AI-assisted evidence synthesis into NPC complication research.

From an implementation perspective, we found that the AdaGrad optimizer facilitated stable training in our screening setting, likely by adapting learning rates to sparse and heterogeneous updates common in medical text classification. We view this as a pragmatic implementation choice to enable convergence rather than a primary scientific contribution. Future studies may benchmark optimizers (e.g., AdamW with learning-rate scheduling) under standardized evaluation protocols to determine whether these training choices materially affect screening reliability and false-negative rates.

Clinically, an accelerated screening-to-synthesis workflow may shorten the time required to update evidence on NPC post-radiotherapy complications, supporting timelier refinement of practice guidance and follow-up strategies. However, the clinical value of speed depends on maintaining adequate sensitivity for study inclusion; thus, we emphasize a risk-aware operating mode in which model-assisted screening is paired with targeted human verification to reduce the likelihood of missing eligible studies, consistent with recent human–LLM collaboration evidence [22]. Separately, our synthesis of frequently reported predictors provides an interpretable summary to support hypothesis generation and risk-aware planning, but any downstream risk model should be interpreted cautiously until validated on independent cohorts.

This study has several limitations. First, our supervised BERT screening approach requires an initial labeled dataset, and labeling quality and inclusion criteria can influence classifier behavior. Second, the workflow is not a fully automated “zero-shot” solution, and recent evidence indicates that human oversight remains necessary to minimize false-negative risk in safety-critical screening contexts [21]. Third, generalizability across institutions, topics, and publication styles remains uncertain because domain shift may affect screening reliability. Future work should benchmark BERT against LLM-based screening under standardized, recall-focused metrics (including confusion matrices and class-wise precision/recall/F1); evaluate external validity using independent NPC datasets; and explore domain-adapted models (e.g., BioBERT) or hybrid strategies, while explicitly quantifying trade-offs among efficiency, sensitivity, and reproducibility.

## 5. Conclusions

In summary, the application of the BERT model for literature screening in this meta-analysis has proven to be highly effective, achieving an AUC of 0.77 and demonstrating the potential to reduce marginal screening workload during downstream application, while not replacing the need for initial manual screening and annotation. The practical utility of this automated workflow was further demonstrated through a downstream meta-analysis, which summarized reported predictive-model performance across the included studies, with random forest models showing higher reported AUC values. These results not only reaffirm the feasibility of integrating advanced NLP technologies with meta-analytical techniques but also highlight the substantial benefits of this approach, including major time savings and enhanced data analysis quality.

In addition to validating the utility of BERT in literature screening, this study provides practical and actionable insights for researchers and clinicians. Researchers can adopt similar approaches to streamline literature reviews in specific medical subfields by fine-tuning BERT models with domain-specific datasets, thereby improving the accuracy of identifying relevant studies. Clinicians, on the other hand, can benefit from these advancements by gaining faster access to synthesized, high-quality evidence, enabling them to make more informed and evidence-based decisions in patient care.

Future research will focus on expanding the application of BERT across diverse medical domains, including the development of disease-specific models tailored to unique clinical and research needs. These efforts aim to further refine automated literature screening, enhance the quality and reliability of medical literature, and significantly increase research efficiency and productivity.

## Figures and Tables

**Table 1:** Classification performance and workflow-level timing results of the BERT-assisted screening process under different optimizer configurations.

Optimizer	AUC	Time Used	Workflow-Level Time Saved (%)
Adam	0.664	1210	99.6%
Adagrad	0.770	1142	99.6%
AdamW	0.590	1159	99.6%

Abbreviations: BERT, bidirectional encoder representations from the transformers. Note: The time saved percentage reflects an illustrative comparison of model execution time relative to manual screening, rather than a directly measured estimate of total screening workload. Due to the large magnitude of manual time compared to the model execution times (1142–1210 s), the percentage reduction rounds to 99.6% for all optimizers.

## Data Availability

The data analyzed in this study were extracted from previously published articles. The extracted datasets and the analytic code used to support the findings of this study are available from the corresponding author upon reasonable request.

## Supplementary Materials

The following supporting information can be downloaded at: https://www.mdpi.com/article/10.3390/reports9010090/s1.

## Abbreviations

AI	artificial intelligence
GPT	generative pre-trained transformer
ROC	receiver operating characteristic
AUC	area under the ROC curve
I^2^	heterogeneity index
τ^2^	between-study variance
Q	Cochran’s Q statistic
