# Supplementary material for: Accelerating Evidence Synthesis: A BERT-Assisted Workflow for Meta-Analyses of Radiotherapy Complications in Nasopharyngeal Carcinoma
Source: Reports (MDPI). 2026 Mar 18;9(1):90. doi: 10.3390/reports9010090 (PMC13030188; doi:10.3390/reports9010090)
Supplement: Supplementary file 1 [file reports-09-00090-s001.zip › reports-4086344-supplementary.pdf]

Supplementary

```python
import pandas as pd

def remove_duplicates(file1, file2, output_file):

    df1 = pd.read_csv(file1)
    df2 = pd.read_csv(file2)

    df1.rename(columns={'Article Title': 'Title'}, inplace=True)
    df2.rename(columns={'Article Title': 'Title'}, inplace=True)

    combined_df = pd.concat([df1, df2], ignore_index=True)

    unique_df = combined_df.drop_duplicates(subset=['Title'], keep='first')

    unique_df.to_csv(output_file, index=False)

    print(f"Duplicate records have been removed, and the results have been saved {output_file}")

file1 = "C:\\Users\\user\\Desktop\\pubmed.csv"
file2 = "C:\\Users\\user\\Desktop\\wos.csv"
output_file = "C:\\Users\\user\\Desktop\\full_paper.csv"

remove_duplicates(file1, file2, output_file)
```

Supplementary Figure S1. The detailed Python data processing code and corresponding pseudocode (Python script for duplicate removal and dataset preparation)

The pseudocode for the Python script

1. **Import necessary library**:
   - Import the pandas library for data manipulation.
2. **Define a function**:
   - Function name: remove_duplicates
   - Input parameters: file1, file2, output_file
3. **Load CSV files into DataFrames**:
   - Read file1 into df1.
   - Read file2 into df2.
4. **Rename columns for consistency**:
   - In df1 and df2, rename the column "Article Title" to "Title" to ensure consistency.
5. **Concatenate DataFrames**:

- Combine df1 and df2 into a single DataFrame, combined_df.

6. **Remove duplicate entries**:

   - Drop duplicate rows in combined_df based on the "Title" column.
   - Keep only the first occurrence of each unique "Title".

7. **Save the cleaned DataFrame**:

   - Save the unique DataFrame to the specified output_file.

8. **Print confirmation message**:

   - Notify the user that duplicate records have been removed and the results have been saved.

9. **Provide file paths and call the function**:

   - Define file paths for file1, file2, and output_file.
   - Call the remove_duplicates function with these file paths.
